# Asthmatic bronchial epithelial cells promote the establishment of a Hyaluronan-enriched, leukocyte-adhesive extracellular matrix by lung fibroblasts

**DOI:** 10.1186/s12931-018-0849-1

**Published:** 2018-08-02

**Authors:** Stephen R. Reeves, Inkyung Kang, Christina K. Chan, Kaitlyn A. Barrow, Tessa K. Kolstad, Maria P. White, Steven F. Ziegler, Thomas N. Wight, Jason S. Debley

**Affiliations:** 1 Division of Pulmonary and Sleep Medicine, Seattle Children’s Hospital, 4800 Sand Point Way NE, Seattle, WA 98105 USA; 2 Center for Immunity and Immunotherapies, Seattle Children’s Research Institute, Seattle, WA USA; 3 Department of Pediatrics, University of Washington, Seattle, WA USA; 4 Matrix Biology Program, Benaroya Research Institute, Seattle, WA USA; 5 Immunology Program, Benaroya Research Institute, Seattle, WA USA

**Keywords:** Asthma, Children, Airway inflammation, Airway remodeling, Epithelial cells, Human lung fibroblasts, Extracellular matrix, Hyaluronan

## Abstract

**Background:**

Airway inflammation is a hallmark of asthma. Alterations in extracellular matrix (ECM) hyaluronan (HA) content have been shown to modulate the recruitment and retention of inflammatory cells. Bronchial epithelial cells (BECs) regulate the activity of human lung fibroblasts (HLFs); however, their contribution in regulating HLF production of HA in asthma is unknown. In this study**,** we tested the hypothesis that BECs from asthmatic children promote the generation of a pro-inflammatory, HA-enriched ECM by HLFs, which promotes the retention of leukocytes.

**Methods:**

BECs were obtained from well-characterized asthmatic and healthy children ages 6–18 years. HLFs were co-cultured with BECs for 96 h and samples were harvested for analysis of gene expression, synthesis and accumulation of HA, and subjected to a leukocyte adhesion assay with U937 monocytes.

**Results:**

We observed increased expression of HA synthases *HAS2* and *HAS3* in HLFs co-cultured with asthmatic BECs. Furthermore, we demonstrated greater total accumulation and increased synthesis of HA by HLFs co-cultured with asthmatic BECs compared to healthy BEC/HLF co-cultures. ECM generated by HLFs co-cultured with asthmatic BECs displayed increased HA-dependent adhesion of leukocytes in a separate in vitro binding assay.

**Conclusions:**

Our findings demonstrate that BEC regulation of HA production by HLFs is altered in asthma, which may in turn promote the establishment of a more leukocyte-permissive ECM promoting airway inflammation in this disease.

**Electronic supplementary material:**

The online version of this article (10.1186/s12931-018-0849-1) contains supplementary material, which is available to authorized users.

## Background

Airway remodeling and inflammatory changes are hallmark pathologic features of asthmatic airway disease [1]. In recent years, there has been a growing appreciation of the central role played by bronchial epithelial cells (BECs) in regulating these processes, giving rise to a new paradigm of disordered epithelial regulation of other important cell types present in the asthmatic airway. BECs represent the interface between the host and the environment and are subject to injury, viral infections, and exposure to aeroallergens that ultimately lead to coordination of the host immune response and/or tissue repair mechanisms [2]. A common pathologic feature of asthma is the accumulation of extracellular matrix (ECM) constituents in the sub-epithelial space, which has been demonstrated in both in vivo animal studies [3–5] and descriptions of human biopsy specimens [6–8]. Recently, work from our group has further characterized these findings utilizing ex vivo co-culture model systems of primary, differentiated human BECs and human lung fibroblasts (HLFs) [9, 10].

Hyaluronan (HA) is a non-sulfated glycosaminoglycan synthesized at the cell membrane through the activity of three hyaluronan synthases (HAS1, HAS2, and HAS3) and is a principal ECM component in all organ systems including the lung [11]. HA is synthesized as a large polymer, high molecular weight HA (HMW-HA, > 250 kDa); however, it can be degraded into low molecular weight HA (LMW-HA) fragments through the activity of hyaluronidases (Hyals), principally Hyal1 and Hyal2, or reactive oxygen species leading to HA turnover [11, 12]. Composition of the HA in tissues, i.e., LMW-HA vs. HMW-HA, is important for its function as differing sizes of HA have been shown to have diverse biological functions and may be either pro- or anti-inflammatory depending on molecular weight and context [11–14].

Elevated levels of HA in brochoalveolar lavage fluid (BALF) obtained from subjects with asthma were initially reported by Sahu and Lynn in 1978 [15]. Subsequently, increased HA levels in airway secretions have been reported in several additional studies and correlate with degree of asthma severity and airway inflammation in humans [16–20] as well as airway inflammation in animal models [3, 4, 21]. In addition to making the ECM more permissible to inflammatory cell infiltration by influencing the viscoelastic properties of the ECM [22], HA can be modified by the binding of various hyaladherins such as the proteoglycan versican (VCAN) or covalently through the activity of tumor necrosis factor stimulated gene 6 (TSG-6), which may also increase its avidity for binding and retaining leukocytes [23].

Previous studies have demonstrated that HLFs isolated from asthmatic donors produce inherently greater amounts of HA in cell culture than HLFs isolated from healthy donors and these differences corresponded to increased expression of HAS2 [14]. Work from our group has also shown that healthy donor-derived HLFs increase *HAS2* expression in response to co-culture with primary differentiated BECs obtained from asthmatic children compared to BECs obtained from healthy children; however, HA production and turnover was not characterized in that study [10]. Thus, the role of BECs in the regulation of HA accumulation in the asthmatic airway remains poorly understood.

To further elucidate the role of BECs in the regulation of HA production by HLFs, we employed an ex vivo model system comprised of primary BECs obtained from well-phenotyped children with or without asthma co-cultured with healthy HLFs. We hypothesized that BECs obtained from an asthmatic donor would drive HLFs to generate an ECM that is more enriched with HA than ECM produced by HLFs that were co-cultured with BECs obtained from healthy donors. Furthermore, we hypothesized that the HA-enriched ECM produced by HLFs co-cultured with BECs obtained from asthmatic donors would bind leukocytes with greater affinity than the ECM generated by healthy BEC/HLF co-cultures.

## Methods

### Subjects

Asthmatic and healthy children between the ages of 6 and 18 years undergoing an elective surgical procedure requiring intubation and general anesthesia were recruited. Asthmatic subjects had at least a one-year history of physician-diagnosed asthma, physician documented wheezing in the prior year, used a short-acting beta-agonist (SABA) greater than or equal to twice a month or were taking a daily controller medication, and were born at 36 weeks gestation or later. Healthy subjects were born at 36 weeks gestation or later, had no history of asthma, reactive airway disease, chronic daily cough, and no history of prior treatment with a systemic or inhaled corticosteroid, SABA, or oxygen. Children with asthma had one or more of the following atopic features: history of positive skin prick test, positive radioallergosorbent testing (RAST) for a common aeroallergen, elevated serum IgE (> 100 IU/mL), history of physician-diagnosed allergic rhinitis, history of physician-diagnosed atopic dermatitis. Written consent was obtained from parents of subjects and assent was obtained for children ≥10 years. The Seattle Children’s Hospital Institutional Review Board approved this study.

### Establishment of bronchial epithelial cell cultures

After the endotracheal tube was secured under general anesthesia, three samples of bronchial epithelial cells (BECs) were obtained from each subject using 4 mm Harrell® unsheathed bronchoscope cytology brushes (CONMED® Corporation). The brush was inserted through an endotracheal tube, advanced until resistance was felt, and rubbed for 2 s against the airway surface as described previously [24]. Cells were seeded onto T-25 cell culture flasks coated with type I collagen and proliferated under submerged culture conditions. Cultures were maintained at 37 °C in an atmosphere of 5% CO_2_ in a humidified incubator in bronchial epithelial growth medium (Clonetics BEGM®; Lonza, Basel, Switzerland) containing gentamicin and amphotericin B, and further supplemented with penicillin-streptomycin (100 μg/ml; Invitrogen®). Fluconazole (25 μg/mL) was added to primary cultures for the first 96 h, after which medium was aspirated and replaced with standard BEGM®. BEGM® was thereafter changed every 48 h until the culture reached ~ 70–90% confluence, after which cells were passaged or cryopreserved.

### Air-liquid Interface (ALI) epithelial cell cultures

BECs were used at passage less than or equal to 3. Cells were seeded onto Corning Costar 12 mm 0.4 μm Transwells® (Corning® Life Sciences) coated with type I collagen at a concentration of 100,000 cells/transwell. Cells were then kept in submerged culture using BEGM in both the apical and well chambers until confluent. Once confluent, cells were then changed to Pneumacult^tm^ ALI Media in the lower well chamber only and the remaining apical media was aspirated. ALI media in the basolateral compartment was changed every other day and cells were differentiated at an ALI for 21 days prior to initiation of cell culture experiments.

### BEC-fibroblast co-cultures

HLFs from a healthy child were obtained from a commercial vender (Lonza, Walkersville, MD) and HLFs from the same passage were used for all co-culture experiments. HLF cultures were established using a Fibroblast Growth Media BulletKit^tm^ (FGM-2). HLFs were seeded at a density of ~ 2500 cells/cm^2^ in 12-well plates coated with type I collagen and incubated for 3–4 days to achieve a confluent monolayer prior to initiation of BEC/HLF co-cultures. Media changes of FGM-2 occurred at 48-h intervals. Beginning with experimental Day 0, the HLF media was replaced with co-culture media (1:1 FGM-2 and PneumaCult ALI Maintenance Media). ALI transwells were placed in co-culture with the HLF cells at experimental Day 0 by transferring the transwell inserts to the well plates containing the established HLF cultures. The transwell inserts containing the BEC ALI culture were in close proximity to the submerged HLF cultures contained in the basolateral chamber and shared the same media. Co-culture media changes occurred daily. Following 96 h of co-culture, BEC ALI transwells were removed and HLF samples were isolated for mRNA expression, analysis of HA content, or leukocyte adhesion studies. HLF and BEC/HLF co-cultures used in adhesion studies were maintained as above with the exception that 10% FBS DMEM was substituted for FGM-2 [25].

### RNA extraction and real-time PCR

Total RNA was isolated from HLFs co-cultured with BECs. Three wells from each experimental condition were harvested and pooled to isolate RNA according to manufacturer recommendations (RNAqueous kit, Ambion®-Applied Biosystems, Austin, TX). RNA concentration and integrity were determined using the Agilent® 2100 Bioanalyzer system and Agilent® RNA 6000 Nano Chips (Agilent® Technologies, Foster City, CA). RNA samples with an RNA integrity number ≥ 8 were reverse-transcribed using the SuperScript® VILO cDNA Synthesis Kit (Life Technologies, Grand Island, NY). Quantitative real-time PCR was performed using validated TaqMan® probes (Life Technologies) for *HAS1, HAS2, HAS3, HYAL1, HYAL2, CD44*, intercellular adhesion molecule 1 (*ICAM*), vascular cell adhesion molecule 1 (*VCAM*), and glyceraldehyde 3-phosphate dehydrogenase (*GAPDH*). Assays were performed using the TaqMan® Fast Advanced Master Mix reagents and the Applied Biosystems StepOnePlus™ Real-Time PCR System (Life Technologies).

### Leukocyte binding assays

Subsets of co-cultured HLFs were used to assess the ability of the secreted ECM to bind U937 cells (ATCC, Mananssas, VA). Cells were washed twice in phenol-free media and re-suspended (3 × 10^6^ cells/mL). Leukocytes were incubated with calcein-AM (0.5 μg/ml; Life Technologies) for 45 min. HLF wells were washed with RPMI. Following this, one mL of the leukocyte suspension was added to the wells and allowed to bind at 4 °C for 90 min. Cultures were washed 5 times in cold RPMI to remove non-adherent cells. Optical densities for well plates and a standard curve were used to calculate the density of adherent cells as previously described [26].

### Immunohistochemistry (IHC)

Sterilized 12-mm round glass coverslips were placed in the bottom of replicate chambers of the 12-well plates prior to seeding the HLFs. Following 96 h of co-culture, the coverslips were carefully removed and stained with biotinylated hyaluronan binding protein (HABP) primary (2.5 μg/ml, Millipore, Burlington, MA) and a streptavidin conjugated Alexa Fluor 568 secondary (1:1000, Thermo Fisher, Waltham, MA). Image quantitation was performed using ImageJ software (NIH, Bethesda, MD). Additionally, U937 cell binding assays were imaged with fluorescent microscopy in calcein-AM-labeled live cell cultures as well following fixation on coverslips. Fixed U937 cell binding assays followed the same IHC protocol as above with the modification of an additional primary antibody against the monocyte marker CD68 (1:200, mouse KP-1 monoclonal, Abcam, Cambridge, UK) and an additional secondary antibody (1:1000, donkey anti-mouse Alexa Fluor 488, Thermo Fisher) at the corresponding steps in the staining protocol.

### Characterization of HA content and fragment sizes

HA quantification was performed using an enzyme-linked sorbent assay (ELSA) for cell layer digests and media samples as previously described [27]. Separate BEC/HLF co-cultures were radiolabeled by the addition of ^3^H acetate 24 h prior to harvesting samples. Radiolabeled macromolecules were recovered and separated from unincorporated isotopic precursors by chromatography on 4 ml columns of sephadex G-50 eluted with 0.1 M Tris acetate, 0.25% Chaps, pH 7.3. Incorporation of ^3^H acetate into HA was measured via chromatography on a sephacryl S-1000 column. A second sample aliquot was measured following digestion of radiolabeled glycosaminoglycans with *Streptomyces* hyaluronidase (2 U/ml, Millipore, Burlington, MA) in Tris/acetate/Chaps for 24 h at 37 °C, followed by rechromatography on sephacryl S-1000. HA was measured as the amount of hyal-sensitive material eluted [25].

### Statistical analysis

For RT-PCR studies, analyses of RT-PCR results were performed using GenEx version 6.0.5 (MultiD Analyses AB, Göteborg, Sweden) based on methods described by Pfaffl [28]. For all other data, the unpaired t-test was used for comparisons that were normally distributed within each subject group. For non-normally distributed data, the Mann-Whitney test was used. Statistical analyses were performed using Prism® 7.0 software (GraphPad Software Inc., San Diego, CA). Statistical significance was set at *P* < 0.05.

## Results

### Subjects

Clinical data obtained from the subjects included in this study is summarized in Table 1. Subjects with and without asthma were similar in age (12.3 ± 3.3 years vs. 11.3 ± 3.4 years, respectively). The asthmatic group contained more males than the healthy group (9 vs. 4). Asthmatic subjects displayed greater markers of atopy including eczema (45%), allergic rhinitis (90%), or positive RAST testing (90%) compared to the healthy control group, which displayed none of these features. Subjects from the asthmatic group also demonstrated higher IgE levels compared to healthy subjects (890 ± 1536 IU/mL vs. 20.9 ± 13.1 IU/mL, *P* < 0.005). Most subjects from the asthmatic group were taking a daily controller medication for their asthma (82%). Lung function testing revealed no differences in FVC % predicted, FEV1% predicted, or FEF 25–75% predicted; however, subjects from the asthmatic group demonstrated significantly reduced FEV1/FVC ratios compared to healthy subjects (82% ± 5% vs. 89% ± 4%, *P <* 0.005). Measurements of fractional exhaled nitric oxide (FENO) trended higher in the asthmatic group; however, this failed to meet statistical significance.

**Table 1:** Subject Characteristics

	Asthmatic Subjects	Healthy Subjects	*P* Value
	*n = 11*	*n = 10*	
Age yrs. (mean ± SD)	12.3 (3.3)	11.3 (3.4)	0.5
Sex (female/male)	2/9	6/4	
Currently using inhaled steroids (%)	9 (82%)		
History of atopy, *n*; (%)	10 (91%)		
Positive RAST, *n*; (%)	10 (91%)		
IgE IU/mL (median ± SD)	890 (1536)	20.9 (13.1)	**0.005**
FVC % predicted (mean ± SD)	99.7 (11.2)	99.8 (13.8)	0.98
FEV_1_/FVC Ratio (mean ± SD)	0.82 (0.05)	0.89 (0.04)	**0.005**
FEV_1_% predicted (mean ± SD)	89.9 (8.2)	100.7 (15.4)	0.24
FEF_25–75_% predicted (mean ± SD)	85.0 (20.4)	104.1 (15.4)	0.08
FENO ppb (mean ± SD)	28.3 (41.1)	9.3 (4.6)	0.15

*RAST* Radioallergosorbent testing, *FVC* Forced vital capacity, *FEV*_*1*_ Forced expiratory volume in 1 s, *FEF*_*25–75*_ Forced expiratory flow between 25 and 75% of expiration

### Co-culture with asthmatic BECs promotes greater HA synthesis by HLFs

**Fig. 1:**
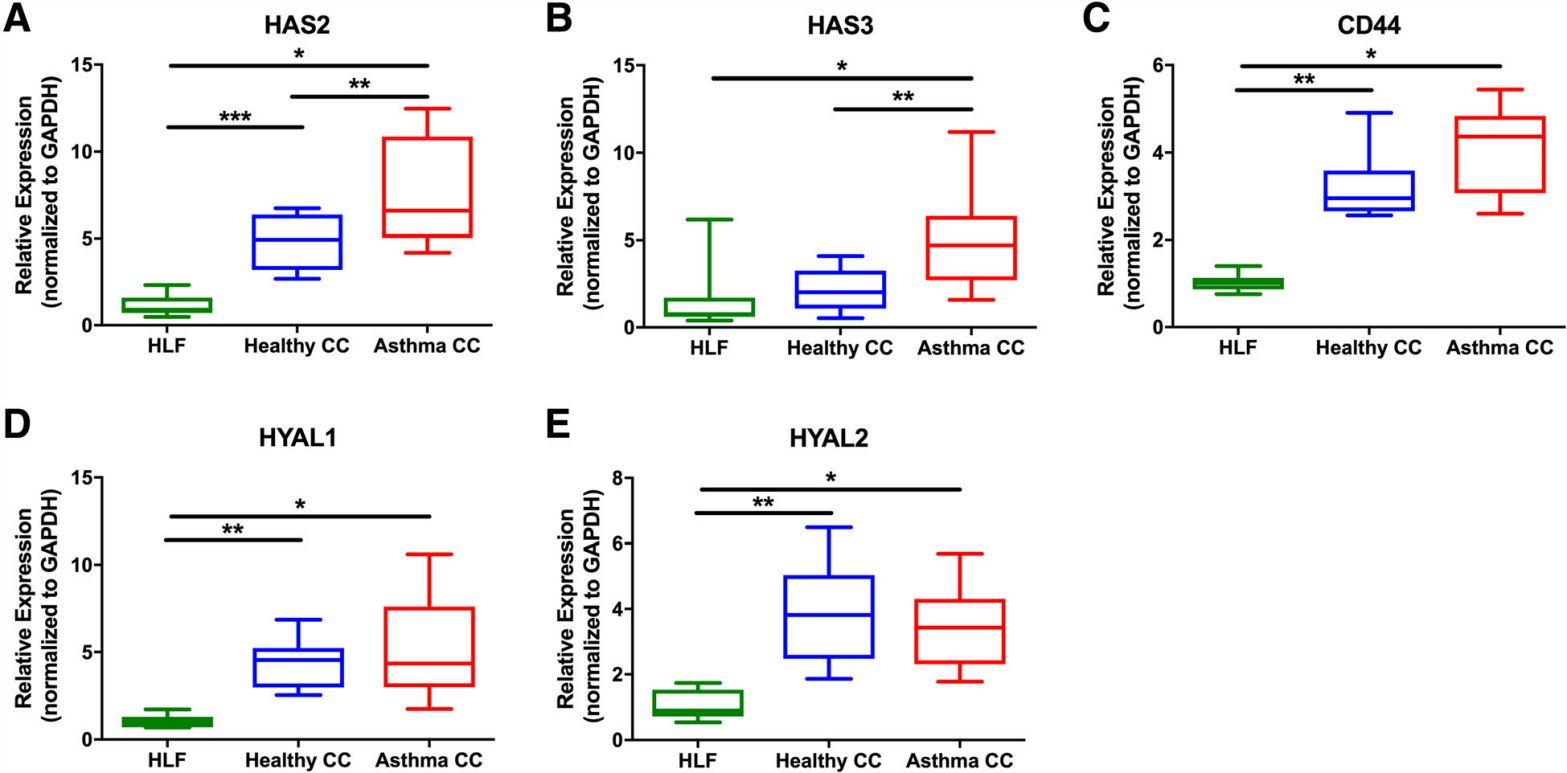
Expression of hyaluronan synthase (HAS) isoforms is increased in human lung fibroblasts (HLFs) alone (*N* = 10) compared to HLFs co-cultured with bronchial epithelial cells (BECs) obtained from healthy (*N* = 10) or asthmatic donors (*N* = 11). **a** *HAS2* expression was significantly greater in HLFs co-cultured with asthmatic BECs compared to HLFs co-cultured with healthy BECs (*P* = 0.02). Expression of *HAS2* was increased in both HLFs co-cultured with healthy BECs and asthmatic BECs compared to HLFs alone (*P* < 0.0001 for both comparisons). **b** Expression of *HAS3* was significantly greater by HLFs co-cultured with asthmatic BECs compared to HLFs co-cultured with healthy BECs (*P* = 0.02). *HAS3* expression was increased in HLFs co-cultured with asthmatic BECs (*P* = 0.005), but not in HLFs co-cultured with healthy BECs compared to HLFs alone. Expression of hyaluronan (HA) degradation enzymes **c** *HYAL1*, **d** *HYAL2*, and co-receptor **e** *CD44* were not significantly different between HLFs co-cultured with healthy BECs and HLFs co-cultured with asthmatic BECs for each comparison. However, expression of *HYAL1*, *HYAL2*, and *CD44* was found to be elevated in HLFs in both healthy and asthmatic co-cultures compared to HLFs alone (*P* < 0.001 for each comparison). Gene expression was normalized to GAPDH and is shown as normalized mean ± SD relative to HLF alone

Comparison of gene expression demonstrated that key HA synthases are upregulated in HLFs as a result of co-culture with asthmatic BECs. HLF expression of *HAS2* was significantly increased in HLFs co-cultured with asthmatic BECs when compared to HLFs co-cultured with BECs from healthy donors (1.6-fold increase, *P* = 0.02; Fig. 1a). HLF expression of *HAS2* was increased in both HLFs co-cultured with healthy BECs and HLFs co-cultured with asthmatic BECs compared to HLFs alone (4.3 fold, *P* < 0.0001; 6.8 fold, *P* < 0.0001 respectively). Expression of *HAS3* by HLFs co-cultured asthmatic BECs was also greater compared to HLFs co-cultured with healthy BECs (2.2-fold increase, *P* = 0.02; Fig. 1b). *HAS3* expression was increased in HLFs co-cultured with asthmatic BECs (3.1 fold, *P* = 0.005), but not in HLFs co-cultured with healthy BECs compared to HLFs alone. Expression of *HAS1* was very low for all HLF samples (CT values > 35), thus making meaningful comparisons difficult. As a result, no significant differences in *HAS1* expression were detected between the two groups (data not shown). In contrast to the finding of increased synthase expression, no differences in the expression of HA degradation enzymes of *HYAL1*, *HYAL2*, or *CD44*, the major HA receptor involved in hyaluronidase activity, were observed between HLFs co-cultured with asthmatic BECs or healthy BECs (*P = NS*, Fig. 1d-f). However, expression of *HYAL1*, *HYAL2*, and *CD44* was found to be elevated in co-cultured HLFs compared to HLFs alone in both healthy co-culture and asthmatic co-culture conditions (*P* < 0.001 for each comparison).

**Fig. 2:**
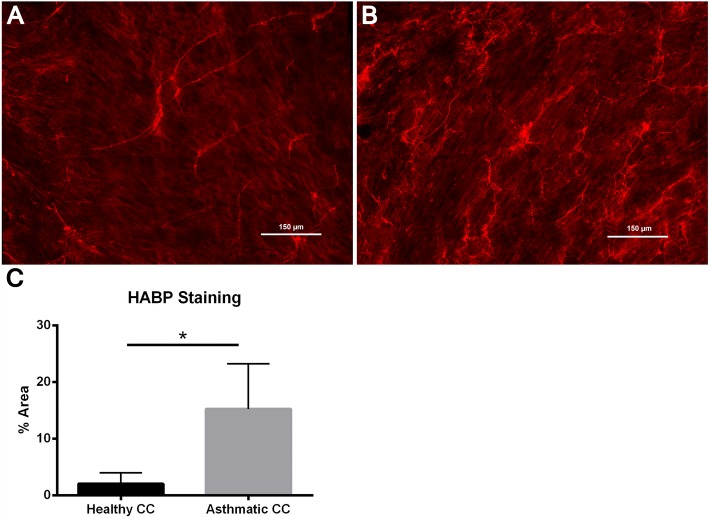
Imaging studies were performed to label HA deposition (red) by HLFs in **a** healthy and **b** asthmatic BEC/HLF co-cultures. Representative images are shown for each condition. **c** Quantitative analysis of HA staining demonstrated that HLFs co-cultured with asthmatic BECs displayed greater HA labeling by % area compared to HLFs co-cultured with healthy BECs (*P* < 0.0001). Data shown as mean ± SD for *N* = 5 replicates/group

**Fig. 3:**
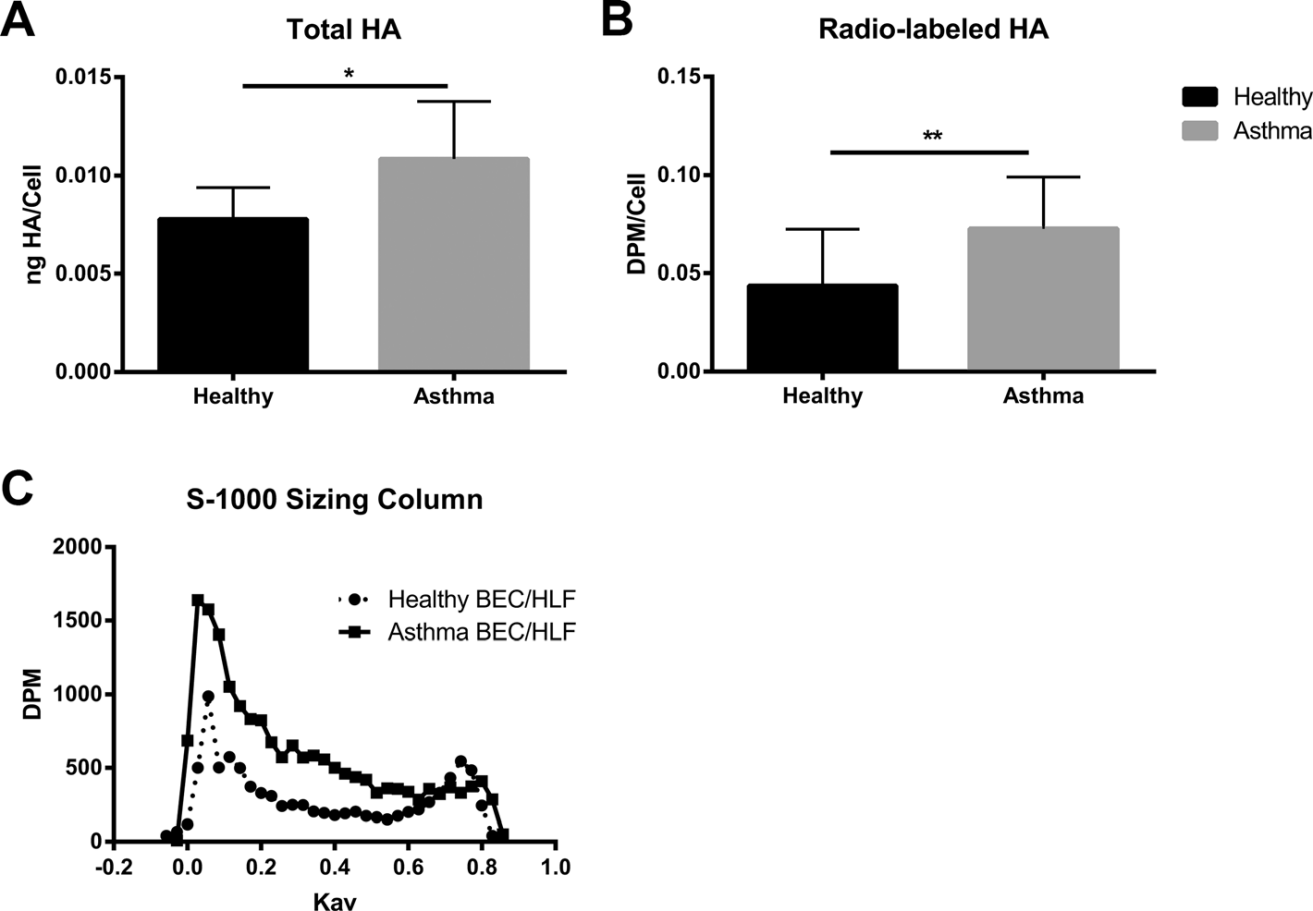
**a** Total HA accumulation as assessed by enzyme linked sorbent assay (ELSA) was greater in HLFs co-cultured with asthmatic BECs compared to HLFs co-cultured with healthy BECs (*P* = 0.04, *N* = 7 /group). **b** Quantitative radiolabeling of HA demonstrated that HLFs co-cultured with asthmatic BECs produced greater total amounts of HA compared to HLFs co-cultured with healthy BECs (*P* < 0.02, *N* = 7 /group). **c** HLFs co-cultured with asthmatic BECs demonstrated a greater abundance of HMW-HA and intermediate weight HA species compared to HLFs co-cultured with healthy BECs. A representative plot of HA-sensitive material as measured by S-1000 size exclusion chromatography is shown. Data is plotted as disintegrations per minute (DPM) vs. the partition coefficient (K_av_)

**Fig. 4:**
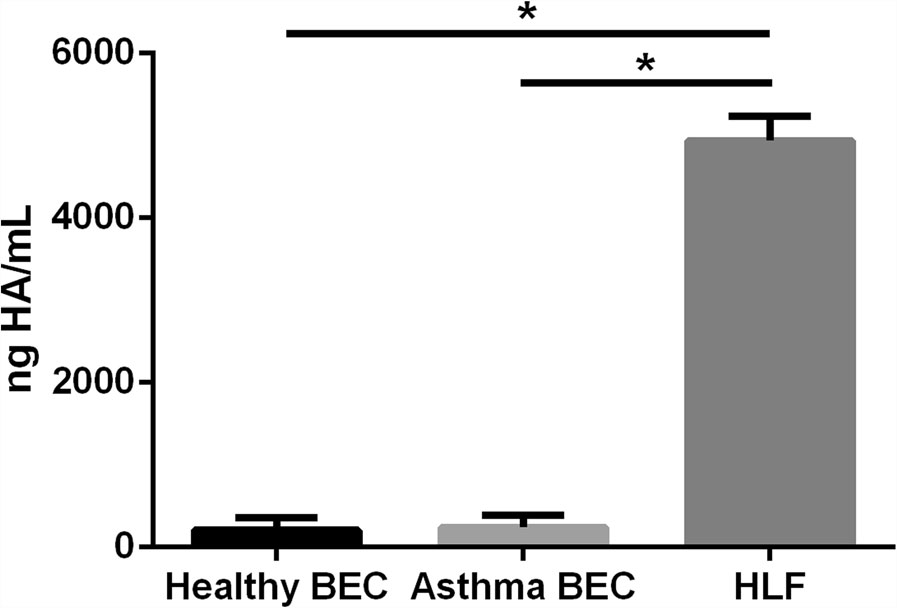
Total HA accumulation in the culture media as assessed by enzyme linked sorbent assay (ELSA) for healthy BECs, asthmatic BECs, and HLFs alone. There was no significant difference in the HA detected in the media between healthy and asthmatic BECs; however, significantly greater HA accumulation was present in the media of HLFs alone compared to either healthy or asthmatic BECs (~ 20 fold; *P* < 0.0001 for both comparisons). Data shown as mean ± SD for (*N* = 7 /BEC groups, *N* = 8 /HLF group)

In addition to enhanced expression of HA synthases, staining for HA by immunohistochemistry also revealed greater amounts of HA staining and HA-cable formation in the HLFs co-cultured with asthmatic BECs compared to HLFs co-cultured with healthy BECs (Fig. 2a, b). Measurements of HA staining by percent area demonstrated greater staining for HA in the HLFs co-cultured with asthmatic BECs compared to HLFs co-cultured with healthy BECs (15.3% ± 1.1% vs. 2.1 ± 0.3%, *P* < 0.0001: Fig. 2c). Representative images of HA staining from both groups are shown. In order to better quantitate differences in HA deposition by HLFs in our co-culture model system, we employed HA ELSA to measure total HA accumulation and radiolabeling techniques to determine de novo HA synthesis. Size exclusion chromatography was used to determine HA fragment size distribution in radiolabeled media samples from both asthmatic and healthy BEC/HLF co-cultures. Compared to healthy BEC/HLF co-cultures, asthmatic co-cultures contained greater total amounts of HA accumulation as measured by ELSA (*P* = 0.04; Fig. 3a). Asthmatic BEC/HLF co-cultures displayed significantly more radiolabeled HA compared to healthy BEC/HLF co-cultures, consistent with enhanced HA synthesis (*P* < 0.02; Fig. 3b). Data is shown normalized to cell count; however, no significant differences in cell number were observed between groups of co-cultured HLFs (Additional file 1: Figure S1). HLFs co-cultured with asthmatic BECs demonstrated a greater abundance of a wide distribution of HA sizes. Both HMW-HA as well as intermediate weight species were consistently increased in HLFs co-cultured with asthmatic BECs compared to HLFs co-cultured with healthy BECs. LMW-HA was found to be elevated as well in some asthmatic BEC/HLF co-cultures; however, this was less consistently observed in the available samples. A representative plot is shown in Fig. 3c. To assess relative contributions of HA production by BECs and HLFs in our co-culture model, we examined the culture media from healthy BECs, asthmatic BECs, and HLFs cultured alone by ELSA (Fig. 4). There was no significant difference in the HA detected in the media between healthy and asthmatic BECs. A significantly greater amount of HA was present in the media of HLFs alone compared to either BEC group (~ 20 fold; *P* < 0.0001 for both comparisons).

### HA-enriched ECM produced by HLFs co-cultured with asthmatic BECs promotes enhanced leukocyte adhesion

**Fig. 5:**
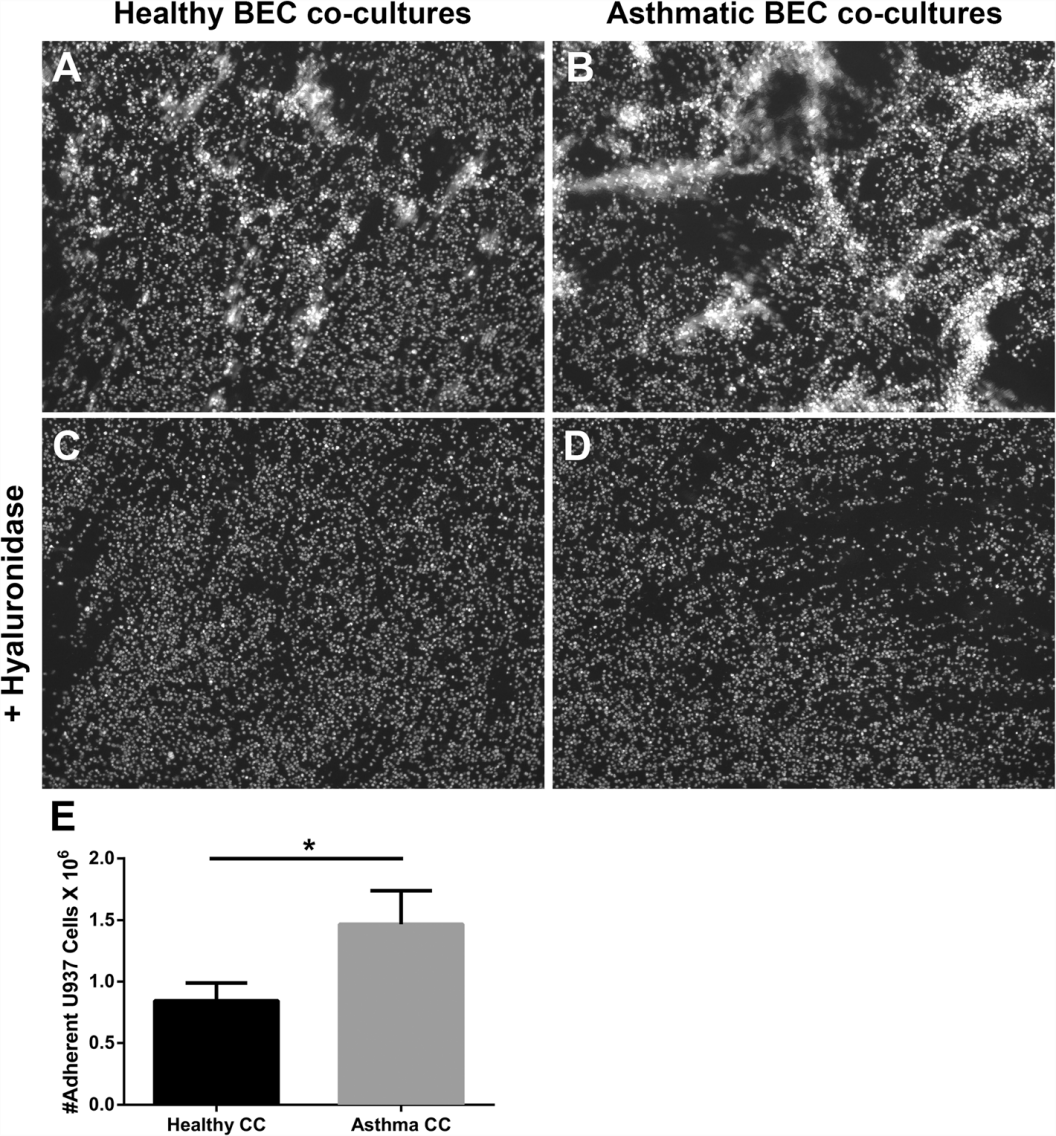
Adhesion of U937 cells to ECM generated by HLFs following 96 h of co-culture with either healthy or asthmatic BECs. Representative images of U937 cell adhesion obtained by fluorescent microscopy depicting binding patterns observed for **a** HLFs co-cultured with healthy BECs and **b** HLFs co-cultured with asthmatic BECs. Pre-treatment with hyaluronidase decreased U937 binding to HLFs co-cultured with either **c** healthy BECs or **d** asthmatic BECs. Quantitative analysis of U937 cell binding using a plate-based assay revealed greater adhesion of U937 cells by HLFs co-cultured with asthmatic BECs compared to healthy BECs (*P* < 0.0001; E). Data shown as mean ± SD for *N* = 10 replicates/group

**Fig. 6:**
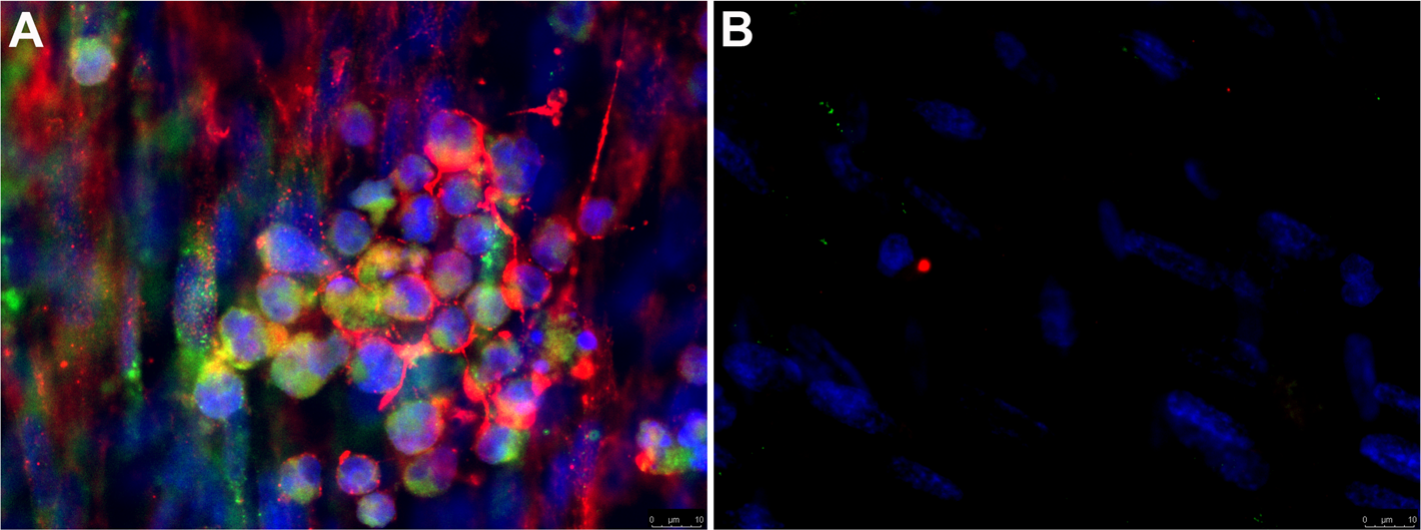
Immunohistochemistry for U937 cell binding assays demonstrating interactions between U937 cells (anti-CD68, green) with HA cables (red) for HLFs co-cultured with asthmatic BECs (Panel **a**). **b** Parallel binding assay performed on HLFs co-cultured with asthmatic BECs after pre-treatment with *Streptomyces* hyaluronidase. Representative images from *N* = 7 replicates

**Fig. 7:**
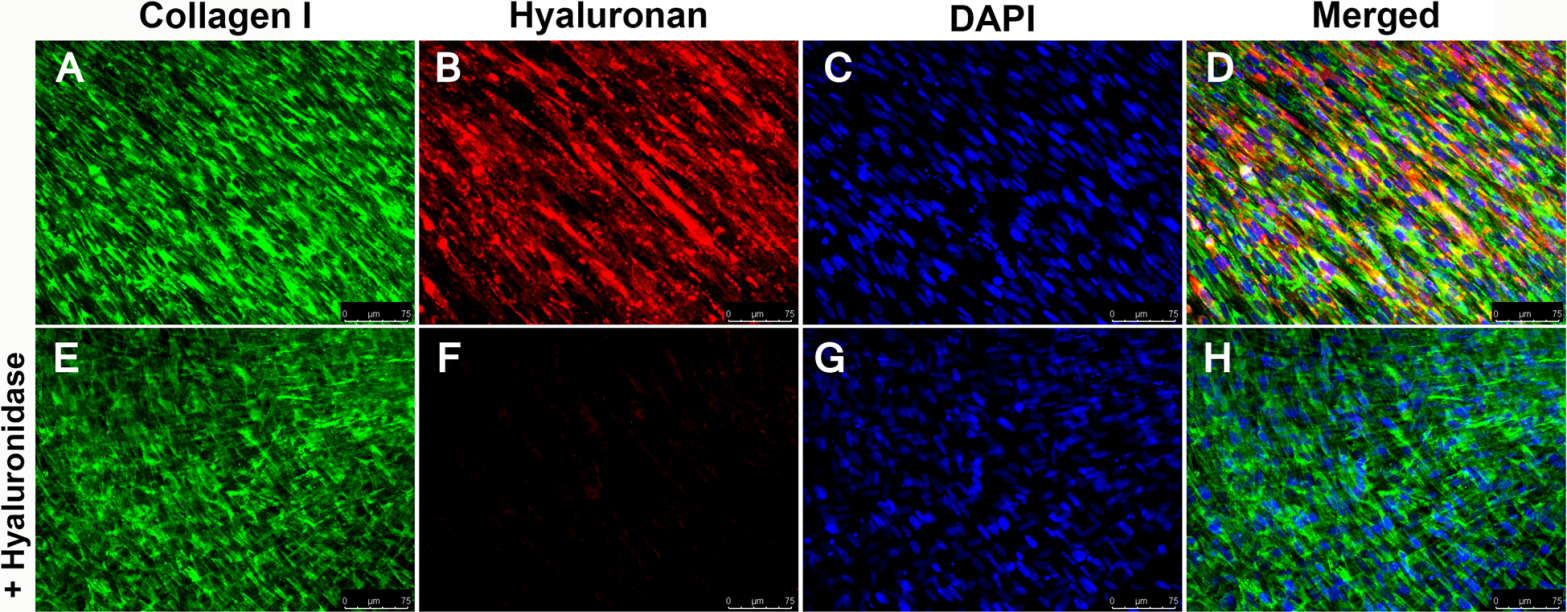
Immunohistochemistry for control HLFs stained for collagen type 1 (**a**, green), hyaluronan binding protein (HABP; panel **b**, red) and DAPI (panel **c**, blue). Panel **d** depicts the merged images. Panels E-H depict staining following pretreatment with hyaluronidase

To determine whether the HA-enriched ECM produced by HLFs co-cultured with asthmatic BECs would promote greater adhesion of leukocytes compared to healthy BEC/HLF co-cultures, we assessed the adhesion of U937 cells via multiple methodologies. To quantitate differences in U937 cell adhesion, we employed a fluorescent label, plate-based assay. The adherence of U937 cells to the ECM generated by HLFs co-cultured with healthy BECs was significantly less compared to HLFs co-cultured with asthmatic BECs (*P* < 0.0001; Fig. 5e). U937 cell adhesion to the HA-enriched ECM produced by co-cultured HLFs was assessed both with and without the addition of Hyal to the media (Fig. 5a-d). We found that U937 cells bound to the ECM layer in aggregates similar to patterns observed in Fig. 2, suggesting that the U937 cells were interacting with HA cables. In wells pre-treated with hyaluronidase, such binding was not observed, suggesting that these cell-ECM interactions are HA-dependent. Separate samples grown on coverslips and stained for HA demonstrated that the U937 cells were indeed binding to the HA cables formed by the co-cultured HLFs. Representative images for asthmatic BEC/HLF co-cultures with and without treatment with hyaluronidase prior to the binding assay are shown in Fig. 6. Treatment with hyaluronidase effectively eliminated staining for HA (shown in red). Binding to the HA cables by U937 cells (stained in green) was not observed after HA digestion with hyaluronidase. To evaluate the specificity of the hyaluronidase for HA compared to fibrillar ECM components such as type 1 and type 3 collagen (COL1, COL3), HLFs were co-labeled for COL1/HABP or COL3/HABP and treated with hyaluronidase for 30 min prior to the staining protocol. Representative images are shown in Fig. 7 (COL1/HABP) and Additional file 2: Figure S2 (COL3/HABP) that demonstrate marked reduction of HA staining following treatment with hyaluronidase without reductions in collagen staining. This further suggests that reduction in U937 binding following hyaluronidase treatment is specific to HA-dependent binding and not other alterations in the ECM. Given that direct interactions between U937 cells and HLFs may also be involved in cell-cell adhesion, we also examined the gene expression of ICAM and VCAM, two major cell adhesion receptors, and found no differences in expression of these adhesion molecules between HLFs co-cultured with healthy or asthmatic BECs (Additional file 3: Figure S3).

## Discussion

In the present study, we have demonstrated in an ex vivo model system that HLFs co-cultured with primary human BECs obtained from asthmatic children produce an ECM that is enriched with HA compared to HLFs that are co-cultured with BECs obtained from healthy children. Furthermore, the HA produced by the HLFs co-cultured with asthmatic BECs is enriched with both HMW-HA and intermediate-sized HA, which may contribute to the enhanced formation of HA cables that was observed in the asthmatic BEC/HLF co-cultures. These higher order HA structures may facilitate the increased ability of the ECM to retain leukocytes and, therefore, could contribute to the inflammation observed in asthma. These findings are in agreement with our previous report of increased HA production in asthmatic BEC/HLF co-cultures [10]. The present work substantially extends our previous findings with additional quantitative and functional studies of leukocyte adhesion demonstrating an important role for the regulation of HLF HA production by BECs.

In addition to size, the structure of HA in the microenvironment can also contribute to a diversity of biological functions. For example, HA can be expressed as a pericellular coat that can regulate cell mobility, differentiation, and proliferation. Alternatively, HA may also be organized into higher order structures such as HA cables that can avidly bind leukocytes [26]. The presence of HA cables has been described in several cellular models including smooth muscle cells, fibroblasts, and multiple types of epithelial cells including keratinocytes, renal tubular epithelial cells, and BECs [26, 29, 30]. While the mechanisms leading to formation of HA cables are not completely understood, several studies have pointed to interactions with proteins and proteoglycans that are known to associate with HA. These HA binding partners are also differentially regulated in the setting of tissue injury and may contribute to the formation of higher order HA structures such as HA cables. Indeed, several studies have demonstrated increased HA cable formation in diverse cellular models of disease, including viral infections, inflammatory bowel disease, and airway inflammation [26, 31]. A common feature of HA cables in each of these studies is the enhanced ability to bind leukocytes. Counter to the ability of LMW-HA fragments to activate leukocytes via receptor-mediated pathways, Day and de la Motte have proposed that the binding of leukocytes by HA cables may, in fact, be a regulatory mechanism to sequester inflammatory cells to limit their interaction with other cell receptors or pro-inflammatory ligands in the environment [32]. The latter may be a very important point in reconciling how HMW-HA and/or higher order HA structures may also contribute to the establishment of chronic inflammatory diseases such as asthma.

Both chronic inflammation and airway remodeling are cardinal features of asthma. Increased deposition of subepithelial proteoglycans and other ECM molecules has been described in several studies of asthma in both adults and children [6–8]. Multiple studies have confirmed that HA is elevated in the airways of subjects with asthma and that this correlates with asthma severity [15, 16, 20, 33]. In a study reported by Liang and colleagues, HLFs isolated from asthmatic subjects produced increased amounts of HA compared to non-asthmatic control subjects, which corresponded to enhanced expression of *HAS2* [14]. Furthermore, the HA produced by the asthmatic HLFs was skewed toward LMW-HA, which the authors proposed contributed to a more pro-inflammatory microenvironment. In a more recent study by Lauer et al., investigators recruited healthy and asthmatic adult subjects for bronchoscopic biopsies, obtained BALF, and performed whole lung allergen challenges. Their findings included an increased amount of heavy chain modified HA (HC-HA) in the thickened basement membrane of asthmatic airways as well as increased HC-HA associated with airway smooth muscle cells and basal epithelial cells in specimens obtained from asthmatic donors. Increased HA in the subepithelial space was associated with increased numbers of leukocytes in the samples collected from asthmatic subjects [33]. Furthermore, the authors reported an increased amount of HA in the BALF from asthmatic subjects that correlated with asthma severity [33]. The finding of increased leukocyte infiltration and greater accumulation of HA in the setting of asthma has also been well characterized in several murine studies of allergen-induced asthma [3–5]. Using an ovalbumin (OVA) sensitization and challenge protocol, Cheng and colleagues demonstrated a time-dependent increase in HA accumulation in the airways of OVA-challenged mice that peaked by day 6 in the lung tissue and day 8 in the BALF [3]. The number of inflammatory cells recovered in the BALF mirrored the presence of HA and were predominately comprised of eosinophils, but also contained increased numbers of lymphocytes and macrophages. Similarly, accumulation of inflammatory cells in the subepithelial space also coincided with the presence of HA, both temporally and anatomically in that study [3]. A follow-up study by the same authors using a cockroach antigen (CRA) sensitization and challenge model of murine asthma recapitulated the findings of the OVA-challenged mice and demonstrated early induction of *HAS1* expression that subsided within the first 24 h of the challenge; however, sustained enhanced expression of *HAS2* was apparent within the first 6 h following the CRA challenge [4]. In a more recent study from our group using a slightly different CRA sensitization protocol, we again demonstrated increased peribronchial and perivascular deposition of HA that was associated with leukocyte infiltration. In addition to the increase in HA staining, we also demonstrated increased staining for the hyaladherin VCAN, which we hypothesized may also play an important role in promoting a more pro-inflammatory environment in asthmatic airway disease during exacerbations [5]. Taken together, these studies demonstrate an important role for the enhanced accumulation of HA in human asthma, which may be an important mechanism for the regulation of inflammatory changes observed in asthmatic airway disease.

A strength of the present study is that our model system uses primary, differentiated BECs obtained from well-phenotyped asthmatic and non-asthmatic children. Using a common primary HLF cell line, we have demonstrated that intracellular crosstalk between BECs and HLFs is differentially regulated in the setting of asthma such that HLFs co-cultured with BECs obtained from asthmatic donors produce an ECM that is enriched with HA and demonstrates greater HA cable formation leading to an enhanced capacity to bind leukocytes in our ex vivo model system. Taken together with the findings of previous studies, our data suggests that BEC signaling is altered in asthma leading to enhanced accumulation of HA-enriched ECM that could predispose to inflammation. In turn, these HA-associated inflammatory changes could directly contribute to the airway obstruction observed in asthmatic airway disease and risk of acute exacerbations in asthmatic subjects.

Despite several strengths, our study does have some limitations. Our cohort is comprised of mostly children with mild or moderate asthma, which is reflective of the patient population at our center. Most of our asthmatic subjects were treated with daily controller medication including inhaled corticosteroids (ICS). Given that the BECs used in this report were studied at passage 2 and had undergone numerous media changes it is unlikely that any residual ICS were present in the media at the time of the experiments. However, we cannot rule out the possibility that persistent steroid effects may be present in our ALI cultures. If present, these effects would likely bias the data toward the null. While our BEC/HLF co-culture model is unique and allows the investigation of BEC influence on HLF behavior, it is difficult to separate out contributions of BECs to the HA content of the media in this model. To address this, we examined the relative contributions from BECs and HLFs cultured alone. A substantially greater amount of HA accumulation was detected in the culture media of the HLFs compared to the BECs. We were not able to detect HA differences in the media between the healthy and the asthmatic BECs. This coupled with the gene expression data would argue that the differences in HA accumulation in our co-culture model are the result of epithelial-derived signaling on the regulation of HA production by the HLFs. Another important limitation of the present study is that we do not yet know what BEC-derived factors account for the alteration in HA accumulation and cable generation by the HLFs. The latter is an active area of investigation in our laboratory and will likely reveal multiple potential targets in this signaling axis for intervention.

## Conclusion

In conclusion, we have demonstrated that primary, differentiated BECs obtained from children with asthma differentially regulate HLFs to produce an ECM that is enriched with HA and has greater affinity for binding leukocytes. Further investigation of the pathways involved in the BEC-HLF crosstalk will likely reveal novel targets or pathways that are involved in these inflammatory changes. A greater understanding of these pathways may lead to novel therapies to treat airway inflammation in asthma.

## Additional files

Additional file 1: **Figure S1.** Cell counts for HLFs co-cultured with healthy or asthmatic BECs (*N* = 7 /group). Data is shown as mean ± SD, no significant differences were observed between the groups. (TIFF 63 kb)

Additional file 2: **Figure S2.** Immunohistochemistry for control HLFs stained for collagen type 3 (A, green), hyaluronan binding protein (HABP; panel B, red) and DAPI (panel C, blue). Panel D depicts the merged images. Panels E-H depict staining following pretreatment with hyaluronidase. (TIF 3321 kb)

Additional file 3: **Figure S3.** Gene expression analysis for (A) ICAM and (B) VCAM by HLFs co-cultured with either healthy or asthmatic BECs (*N* = 10 /group). Gene expression was normalized to GAPDH and is shown as normalized mean ± SD relative to the healthy controls. (TIFF 241 kb)

## Abbreviations

ALI: Air-liquid interface
BALF: Brochoalveolar lavage fluid
BECs: Bronchial epithelial cells
BEGM: Bronchial epithelial growth medium
COL1: Collagen type 1
COL3: Collagen type 3
ECM: Extracellular matrix
FEF_25–75_: Forced expiratory flow between 25 and 75% of FVC
FENO: Fraction of exhaled nitric oxide
FEV_1_: Forced expiratory volume in 1 s
FVC: Forced vital capacity
GAPDH: Glyceraldehyde 3-phosphate dehydrogenase
HABP: Hyaluronan binding protein
HAS: Hyaluronan synthase
HLFs: Human lung fibroblasts
HMW-HA: high molecular weight hyaluronan
Hyal: Hyaluronidases
ICAM: Intercellular adhesion molecule
ICS: Inhaled corticosteroids
IgE: Immunoglobulin E
LMW-HA: Low molecular weight hyaluronan
RAST: Radioallergosorbent testing
TSG-6: Tumor necrosis factor stimulated gene 6
VCAM: Vascular cell adhesion molecule
VCAN: versican
