# Supplementary figures and images for: Asthmatic bronchial epithelial cells promote the establishment of a Hyaluronan-enriched, leukocyte-adhesive extracellular matrix by lung fibroblasts
Source: Respir Res. 2018 Aug 2;19:146. doi: 10.1186/s12931-018-0849-1 (PMC6090698; doi:10.1186/s12931-018-0849-1)

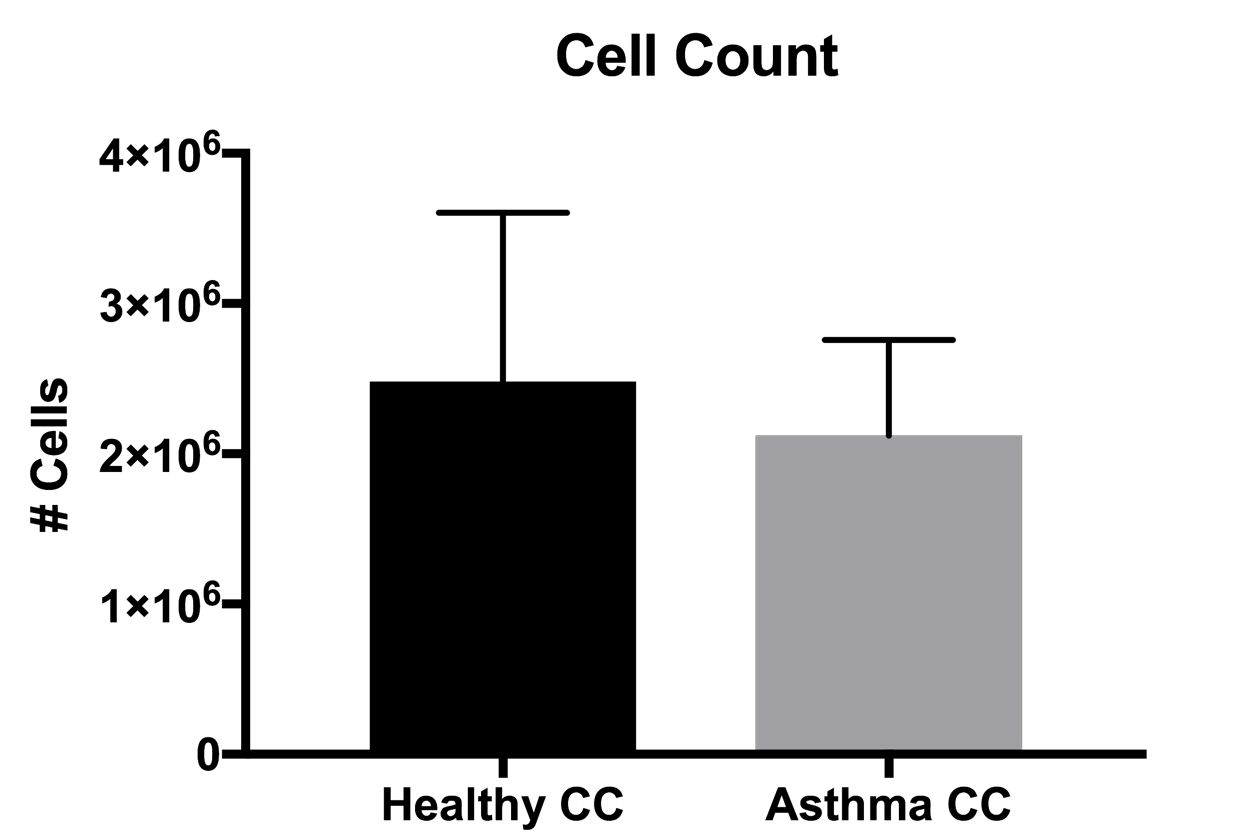

Supplement: Supplementary file 1 — Figure S1. Cell counts for HLFs co-cultured with healthy or asthmatic BECs (N = 7 /group). Data is shown as mean ± SD, no significant differences were observed between the groups. (TIFF 63 kb) [file 12931_2018_849_MOESM1_ESM.tiff]

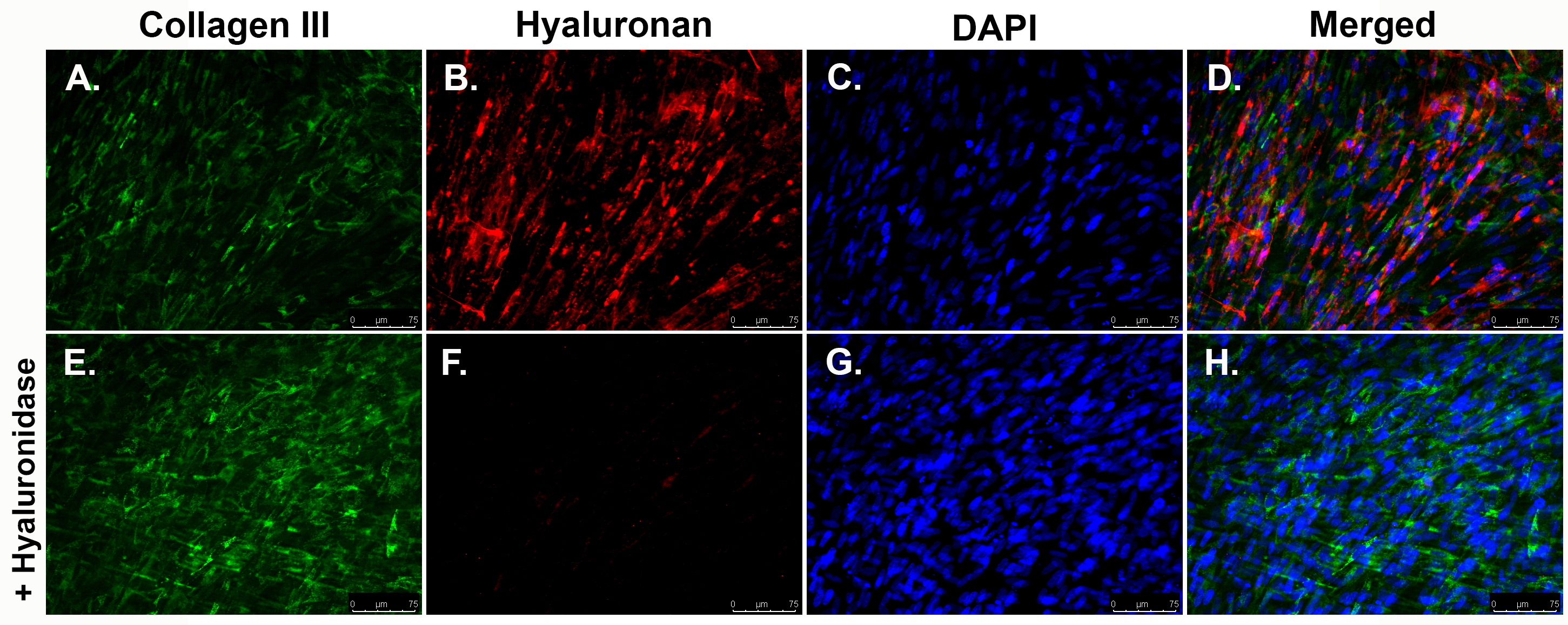

Supplement: Supplementary file 2 — Figure S2. Immunohistochemistry for control HLFs stained for collagen type 3 (A, green), hyaluronan binding protein (HABP; panel B, red) and DAPI (panel C, blue). Panel D depicts the merged images. Panels E-H depict staining following pretreatment with hyaluronidase. (TIF 3321 kb) [file 12931_2018_849_MOESM2_ESM.tif]

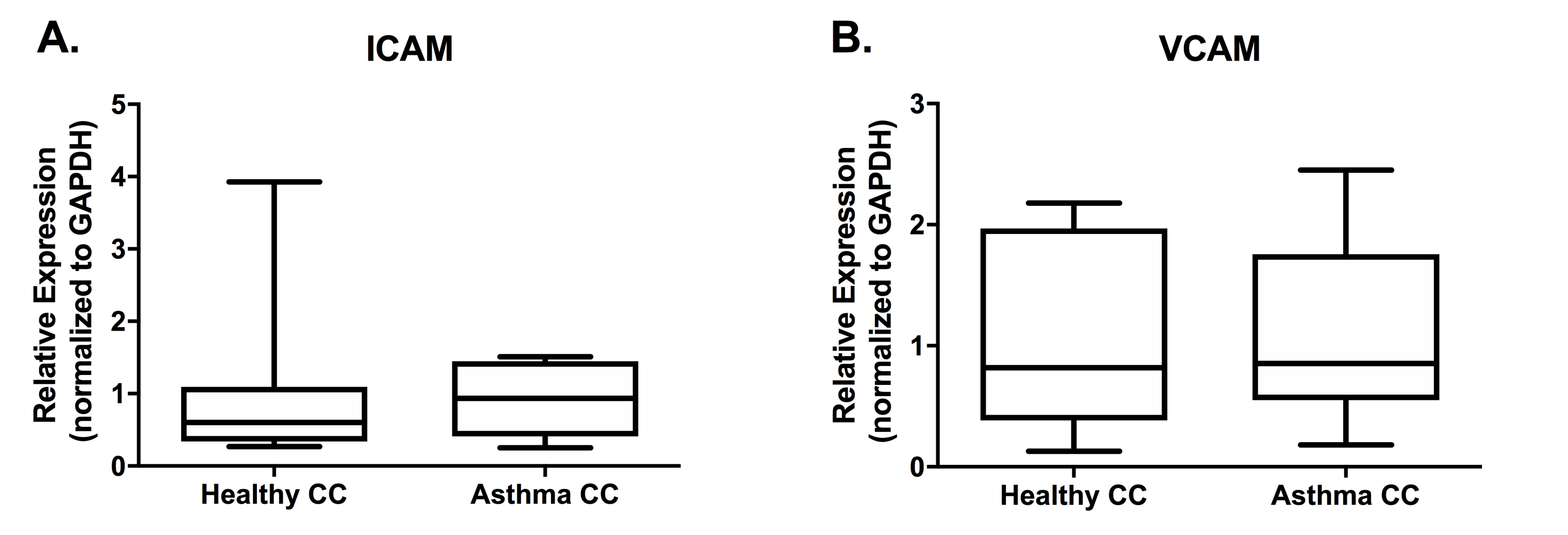

Supplement: Supplementary file 3 — Figure S3. Gene expression analysis for (A) ICAM and (B) VCAM by HLFs co-cultured with either healthy or asthmatic BECs (N = 10 /group). Gene expression was normalized to GAPDH and is shown as normalized mean ± SD relative to the healthy controls. (TIFF 241 kb) [file 12931_2018_849_MOESM3_ESM.tiff]
